# Lung fibrotic tenascin-C upregulation is associated with other extracellular matrix proteins and induced by TGFβ1

**DOI:** 10.1186/1471-2466-14-120

**Published:** 2014-07-26

**Authors:** Susanna Estany, Vanesa Vicens-Zygmunt, Roger Llatjós, Ana Montes, Rosa Penín, Ignacio Escobar, Antoni Xaubet, Salud Santos, Frederic Manresa, Jordi Dorca, Maria Molina-Molina

**Affiliations:** 1 Respiratory Research Group, IDIBELL, University of Barcelona, Barcelona, Spain; 2 Unit of Interstitial Lung Diseases, Department of Respiratory Diseases, University Hospital of Bellvitge, Barcelona, Spain; 3 Department of Pathology, University Hospital of Bellvitge, Barcelona, Spain; 4 Department of Thoracic Surgery, University Hospital of Bellvitge, Barcelona, Spain; 5 Department of Pulmonology, Hospital Clinic, Barcelona, Spain; 6 CIBER, National Research Consortium CIBER of Respiratory Diseases (CIBERES), c. Feixa Llarga, s.n. 08907 L'Hospitalet de Llobregat, Barcelona, Spain

**Keywords:** Extracellular matrix, Idiopathic pulmonary fibrosis, Glycoproteins, Tenascin-C

## Abstract

**Background:**

Idiopathic pulmonary fibrosis (IPF) is a progressive parenchymal lung disease of unknown aetiology and poor prognosis, characterized by altered tissue repair and fibrosis. The extracellular matrix (ECM) is a critical component in regulating cellular homeostasis and appropriate wound healing. The aim of our study was to determine the expression profile of highlighted ECM proteins in IPF lungs.

**Methods:**

ECM gene and protein expression was analyzed by cDNA microarrays, rt-PCR, immunohistochemistry and western-blot in lungs from idiopathic pulmonary fibrosis (IPF), hypersensitivity pneumonitis (HP), categorized as chronic (cHP) and subacute (saHP), and healthy lung tissue. Primary fibroblast cultures from normal subjects and fibrotic patients were studied to evaluate tenascin-C (TNC) synthesis.

**Results:**

A total of 20 ECM proteins were upregulated and 6 proteins downregulated in IPF. TNC was almost undetected in normal lungs and significantly upregulated in fibrotic lungs (IPF and cHP) compared to saHP. Furthermore, it was located specifically in the fibroblastic foci areas of the fibrotic lung with a subepithelial gradient pattern. TNC levels were correlated with fibroblastic foci content in cHP lungs. Versican and fibronectin glycoproteins were associated with TNC, mainly in fibroblastic foci of fibrotic lungs. Fibroblasts from IPF patients constitutively synthesized higher levels of TNC than normal fibroblasts. TNC and α-sma was induced by TGF-β1 in both fibrotic and normal fibroblasts. TNC treatment of normal and fibrotic fibroblasts induced a non-significant increased α-sma mRNA.

**Conclusions:**

The difference in ECM glycoprotein content in interstitial lung diseases could contribute to the development of lung fibrosis. The increase of TNC in interstitial areas of fibrotic activity could play a key role in the altered wound healing.

## Background

Idiopathic pulmonary fibrosis (IPF) is the most common and lethal lung fibrotic process [1]. The histological defined pattern is the usual interstitial pneumonia (UIP) characterized by the loss of epithelial structures, the appearance of interstitial fibrosis, microscopic honeycombing and focal areas of fibroblast-myofibroblast aggregates termed “fibroblastic foci”. These foci are associated with poor prognosis of disease and contain several hallmarks of the reactive stroma [2]. This stroma exhibits an excessive deposition of extracellular matrix (ECM) components, such us collagen I, collagen III and structural glycoproteins and proteoglycans.

During the last few years, there has been an increased interest in the role of glycoproteins and proteoglycans in the pathological process of altered wound healing. Tenascin C (TNC) is a large hexameric ECM glycoprotein that is specifically and transiently expressed upon tissue injury; it is activated after local injury and down-regulated when tissue repair or scarring is concluded [3]. TNC has a role in cell adhesion, fibroblast migration, and other processes related to tissue remodeling and wound healing [4,5]. Paron and col. [6] showed that TNC was able to support and promote growth and migration of pancreatic cancer cells. Other authors have described the association of TNC expression with the progression and aggression of cancer [7].

Versican (VCAN), a chondroitin sulfate proteoglycan, participates in cell adhesion, proliferation, migration and angiogenesis and hence plays a central role in tissue morphogenesis and maintenance [8,9]. In addition, this protein contributes to the development of a number of pathologic processes including atherosclerotic vascular diseases, cancer, tendon remodel ng and central nervous system injury [10].

Fibronectins (FNs) are multifunctional glycoproteins found in the ECM of several tissues and plasma. Cellular FN, a multimeric form synthesized by mesenchymal, epithelial, and inflammatory cells, is deposited in ECM fibrils and contains variable proportions of the extra type III domains A and B (EDA and EDB). Muro *and col*. [11] showed that EDA-containing FN is essential for the lung collagen deposition after bleomycin-induced lung injury in rats, which suggests that EDA-FN would play a critical role in experimental induced tissue fibrogenesis.

ECM provides not only structural and mechanical support, but it is also implicated in important biological functions during tissue remodeling. The abnormal wound repair and pro-fibrotic activity seem to be crucial in the progression of lung fibrosis. Since the ECM glycoproteins are important in the wound healing process, these proteins could be crucial for the development of lung fibrosis after tissue injury. For this reason, we hypothesized that a better characterization of the ECM protein expression in IPF lungs could provide relevant information to understand the progression of this lethal interstitial lung disease and to open new targets for therapeutic approaches. The aim of our study was to analyze the lung ECM proteins in IPF.

## Methods

### Human tissue samples

Pathological interstitial lung tissue was obtained by lung video-assisted thoracoscopic surgery from patients undergoing diagnostic evaluation at the Unit of Interstitial Lung Diseases of the Respiratory Diseases department from Bellvitge University Hospital (Hospitalet del Llobregat, Spain) and the Department of Pneumology of Hospital Clínic de Barcelona (Barcelona, Spain). The diagnosis of IPF, chronic hypersensitivity pneumonitis (cHP) and subacute hypersensitivity pneumonitis (saHP) was performed following the ATS/ERS guidelines [1,12], using two biopsies from different lung lobes after the histological evaluation of two expert pathologists in interstitial lung disease. All cases of IPF fulfilled documented criteria for a diagnosis of UIP, and patients with cHP presented different degrees of lung fibrosis. In those cases, a scoring system to determine the extent of fibroblastic foci was undertaken independently by two pathologists according to the Brompton scoring method [2], blind to the clinical or biomolecular data. Healthy lung tissue was obtained from individuals undergoing surgical treatment for spontaneous pneumothorax, without clinical or hystopathological evidence of pulmonary disease.

The Ethics Committee of Bellvitge University Hospital and Hospital Clinic approved the study. Written informed consent was previously obtained from all individuals according to institutional guidelines.

### Primary human fibroblast studies

Primary fibroblasts were isolated from normal and IPF lung tissues, as previously described [13]. Briefly, 1 mm^2^ fragments of tissue were incubated under sterile conditions in Dulbecco’s modified Eagle’s media (DMEM) (Gibco, Grand Island) supplemented with 10% fetal bovine serum (FBS) (Gibco), 100 IU/ml penicillin (Gibco), 100 μg/ml streptomycin (Gibco) and 2 μg/ml amphotericin B (Sigma). Cell cultures were kept in a 5% CO_2_ humidified atmosphere at 37°C. For all experiments, primary lung fibroblasts were used between passages 3 and 5, and plated at a density of 2 × 10^5^ cells/well in 6-well culture plates. When cell culture reached 70% of confluence, cells were serum starved in DMEM for 24 hours prior to stimulation with TNC (Millipore, Bedford, MA) at a final concentration of 10 μg/ml or TGF-β1 at 5 ng/ml (R&D Systems, Minneapolis, MN), for 4 and 24 hours.

### RNA extraction and retrotranscriptase

Lung samples and fibroblast cells were lysed in Trizol (Invitrogen, Carlsbad, CA) and total RNA was extracted. After the ethanol precipitation, RNAs were cleaned up and genomic DNA was eliminated using the RNAeasy plus Mini kit (Qiagen, Valencia, CA). RNA concentration and purity was analyzed by UV-spectrophotometry (NanoDrop, Thermo Scientific). Absorbance ratio A_260_:A_230_ and A_260_:A_280_ were greater than 1.7 and 2.0, respectively. If not, an extra ethanol precipitation was performed. For microarray experiments, ribosomal RNA band integrity was verified on Agilent BioAnalyzer 2100 using an Eukaryote Total Nano Assay. cDNA was synthesised with one microgram of RNA by High Capacity cDNA Reverse Transcription kit (Applied Biosystems, Foster City, CA).

### Oligonucleotide microarray experiments

The normal RNA pool and the IPF RNA pool were performed using equal quantities of RNA of 7 normal subjects and 12 patients with IPF, respectively. cDNA was synthesized with one microgram of each pool. cDNA microarray of extracellular matrix proteins (Human Extracellular Matrix and Adhesion Molecules PCR Array, SABioscience) was made with both pools according to the manufacturer’s recommendation. The results were analyzed by the ΔΔCt method.

### Reverse transcription real-time PCR

Lungs from 22 patients with fibrotic interstitial lung disease (ILD) (17 patients with IPF and 5 patients with cHP), 4 patients with an inflammatory ILD (such as saHP) and 7 normal subjects were analyzed by quantitative reverse transcription-polymerase chain reaction (qPCR). qPCR was performed with taqMan gene expression master mix (Applied Biosystems) for TNC, VCAN and EDA-FN. The results were analyzed by the ΔΔCt method, normalized with two housekeeping genes (Eukaryotic 18S rRNA and DNA-directed RNA polymerase II) and relative to a common calibrator for every plate. All samples were run in triplicate.

For primary fibroblast experiments, qPCR for TNC and α-sma normalized with these two housekeeping genes were done in the same way.

### Western blot

Small pieces of lung samples were homogenized using a lysis buffer (50 mM Tris, 150 mM NaCl, 1% Igepal, 5 mM EDTA, 1 mM DTT, 1/1000 protease inhibitor) for 30 min at 4°C, in an orbital mixer. Protein concentration was determined using total protein kit quantification (Sigma-Aldrich).

100 μg protein samples were separated on mini-protean TGX, 4-15% gel (Bio-Rad, Hercules, CA) in TRIS/GLICINE/SDS Running Buffer (Bio-Rad) and transferred to nitrocellulose membrane (Bio-Rad). Afterwards, they were probed with a mouse monoclonal for TNC (1/200; Abcam, Cambridge, UK), α-sma (1/1000, 1A4, Sigma Aldrich, St Louis, MO), and β-actin or β-tubulin (1/10000, Sigma Aldrich, St Louis, MO) overnight. Immunoblots were incubated with peroxidase-conjugated rabbit anti-mouse (1/1000 dilution, DakoCytomation) for 1 hour and developed using SuperSignal West Pico Chemiluminescent Substrate (Pierce, Rockford, IL). For Western blot analysis, the densitometry unit of the protein expression in control cells was assigned as 1 after normalized with β-actin.

### Immunohistochemistry

Lung samples were fixed in formaldehyde, embedded in paraffin and serial sections were prepared for cell (alpha-smooth muscle actin-α-sma cells) and ECM glycoprotein staining. Tissue sections were deparaffinized, rehydrated and then antigen was retrieved by being boiled in Tris-EDTA buffer (pH 9.0) for 5 min. Samples were blocked with 0,3% H_2_O_2_ for 1 h with avidin/biotin blocking kit (vector). Immunohistochemistry was carried out using primary antibody of tenascin C (1/300, Abcam), versican (1/250, Lifespan) and fibronectin domain EDA (1/400, Abcam), followed by biotin-avidin/peroxidase (Vectastain Elite ABC kit) incubation. Alpha-smooth muscle actin (α-sma) (RTU Flex, Dako) was performed with Autostainer Link Instrument, using EnVision Flex+, mouse, and high pH (Link) (Dako), according to the manufacturer’s recommendation. All sections were counterstained with haematoxylin.

### Statistical methods

Data were analyzed with PASW Statistics version 18. Relative PCR values were analyzed by Kruskal-Wallis test to compare more than two groups, and Mann–Whitney U-test or Wilcoxon Signed Rank Test when comparing two independent or related samples, respectively. ANOVA with post-hoc analysis, corrected with Scheffé’s method, was used to compare the TNC, VCAN, and FN gene expression pairwise between different groups. The relationship between the semiquantitative% fibroblast-foci score, FVC and DLCO, and the level of TNC in fibrotic lungs was analyzed using the Spearman rank correlation coefficient. Two-tailed p-values lower than 0.05 or 0.01 were considered significant. To evaluate linear dependency of numerical variables, Pearson correlation coefficient was calculated.

## Results

### Subjects

The clinical and functional characteristics of the patients are presented in Table 1. The mean age for IPF group was similar to the cHP group, but the saHP and the normal groups were younger (p = 0.03 and p < 0.001, respectively). Smoking history was present in all studied groups. Sex distribution was similar among all the groups, with a predominance of male versus female. The IPF subjects showed the lowest value of forced vital capacity (FVC) (63% ± 14.6%) when compared with normal group (91.8% ± 2.4%, p < 0.001), cHP (68% ± 16%, p = 0.5) and saHP patients (72% ± 17.5%, p = 0.42). The diffusion lung capacity for carbon monoxide (DLCO) was significantly lower in IPF patients (43.5% ± 15.5%), as compared to normal subjects (90.4% ± 3.5%, p < 0.001), but resulted similar to cHP patients (45.2% ± 18.5%, p = 0.73) and saHP group (48.4% ± 17.5%, p = 0.8). Changes in DLCO and FVC over 6 months were significantly different between IPF and saHP (Table 1).

**Table 1:** Characteristics of the study groups

	**Control**	**IPF**	**cHP**	**saHP**
**Number of subjects**	**7**	**17**	**5**	**4**
**Age (years)**	40,2 ± 9,8*	**62,5 ± 5**	**59,4 ± 8,2**	53,5 ± 0,7*
**Male/Female**	**5/2**	**12/5**	**4/1**	**2/2**
**Smoking (no/Ex/C)**	**4/1/2**	**6/9/2**	**2/2/1**	**1/3/0**
**FVC% pred**	91,8 ± 2,4*	**63,3 ± 14,2**^**#**^	**73 ± 16,5**	**72 ± 9,9**
**DLCO% pred**	90,4 ± 3,5*	**43,7 ± 15**^**#**^	**45,2 ± 18,5**^**#**^	**48,4 ± 17,5**
**%FVC change**		**- 3,2 ± 6,2**	**- 1,9 ± 3,1**	- 0,1 ± 2,1*
**%DLCO change**		**- 2,9 ± 5,9**	**- 1,1 ± 3,5**	+ 0,3 ± 4,5*

Data are presented as mean ± SD. Results were analyzed using t-student test for parametric values or Mann–Whitney Rank Sum test for non-parametric values. *p < 0,05: significance difference compared with the IPF group; # p < 0,05: significance difference compared with the control group.

No: never smoking; Ex: ex-smoker; C: current smoker; FVC: forced vital capacity; DLCO: diffusion lung capacity for carbon monoxide; IPF: idiopathic pulmonary fibrosis; cHP: chronic hypersensitivity pneumonitis; saHP: subacute hypersensitivity pneumonitis. %FVC change: Absolute change in percent-predicted FVC over 24 weeks. %DLCO change: Absolute change in percent-predicted DLCO over 24 weeks.

### Tenascin C upregulation in lung fibrosis

**Figure 1:**
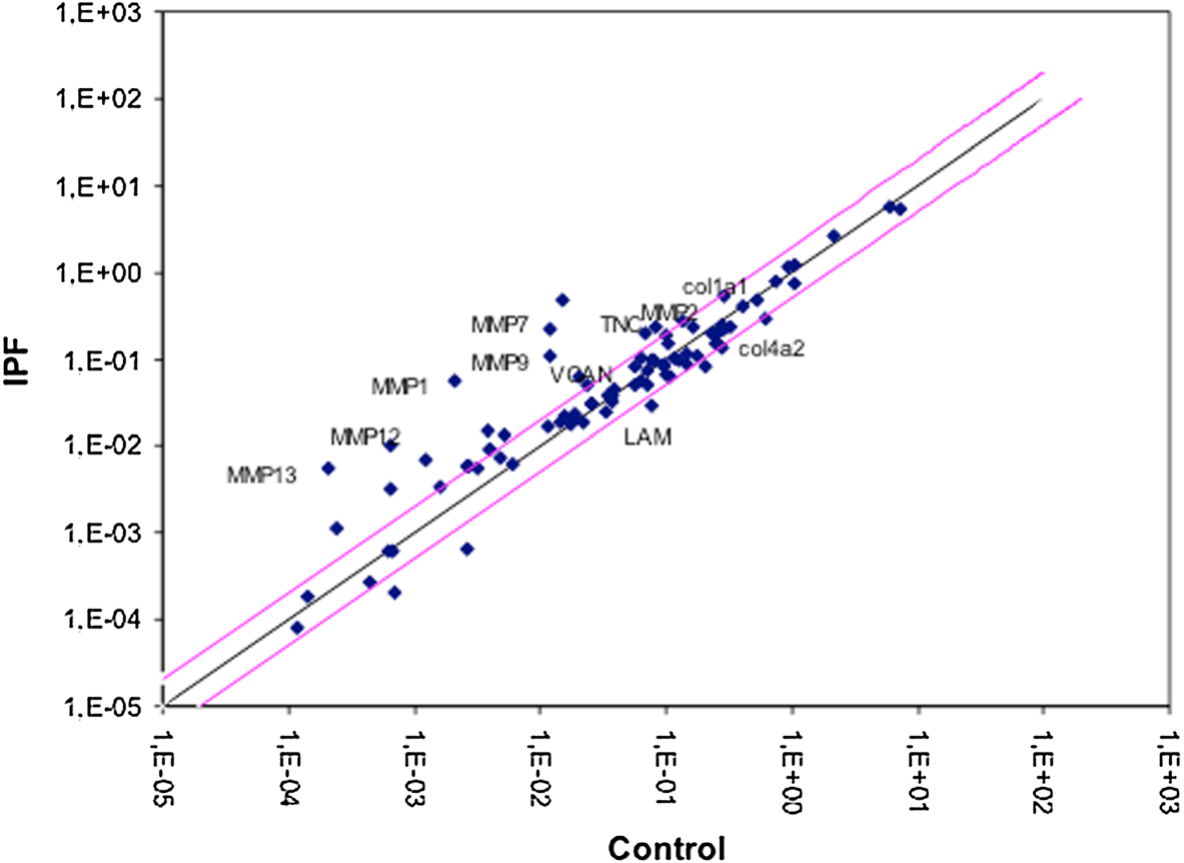
**cDNA microarray of ECM proteins reveals genes that are more than 2-fold up- and down-regulated in IPF.** Scatter plot represents gene expression of each protein in IPF lungs (n = 17) versus control lungs (n = 7). Genes that are more than 2-fold up- or down-regulated in IPF are outside the red line. Detailed values for each protein are included in Additional file 1.

**Figure 2:**
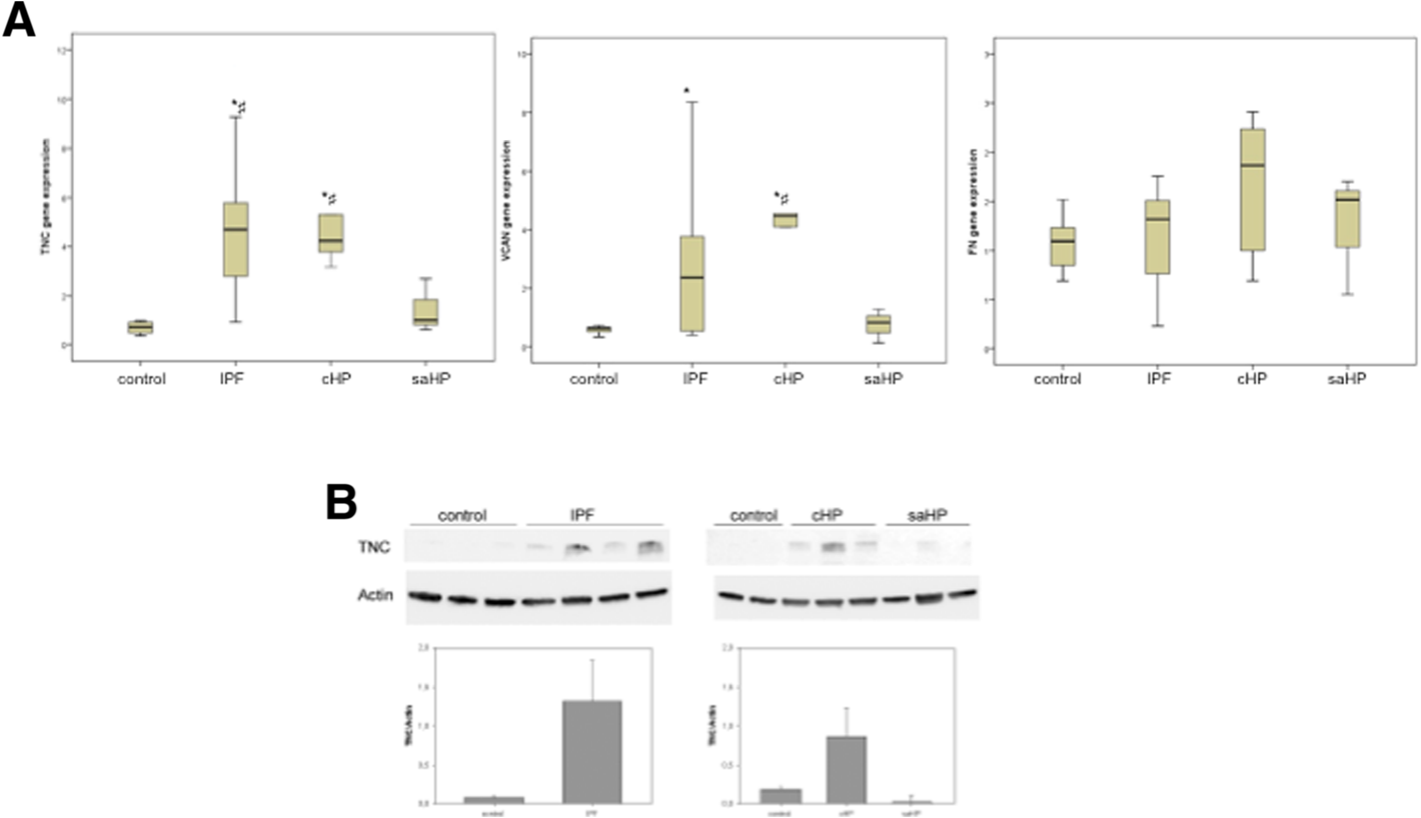
**Glycoprotein synthesis in lung fibrosis. A)** TNC, VCAN and FN gene expression in IPF (n = 17), cHP (n = 5), saHP (n = 4) and control lungs (n = 7). The bottom, middle band and top of the box are the first quartile, median and third quartile, respectively. TNC was significantly increased in IPF and cHP lungs compared with control (*p < 0.05) and saHP lungs (#p < 0.05). VCAN was higher in IPF and cHP compared with control lungs (*p < 0.05), and this increase was also significantly different in cHP compared with saHP lungs (#p < 0.05). No significant differences among groups were found in FN gene expression. **B)** Western-blotting showing an increase of TNC protein in IPF and cHP lungs compared with normal and saHP lungs. IPF: idiopathic pulmonary fibrosis, cHP: chronic hypersensitivity pneumonitis, saHP: subacute hypersensitivity pneumonitis, TNC: tenascin C, VCAN: versican, FN: fibronectin.

**Figure 3:**
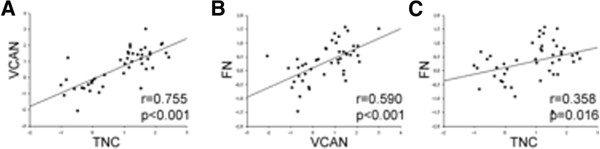
**Relationship among TNC, VCAN and FN gene expression.** Pearson correlation shows a strong association between **A)** VCAN and TNC (r = 0.755, p = 0.001), **B)** FN and VCAN (r = 0.59, p = 0.001), **C)** FN and TNC (r = 0.35, p = 0.01). TNC: tenascin C, VCAN: versican, FN: fibronectin.

To identify the differences in the ECM protein expression profile of fibrotic lungs that differ from normal lungs, cDNA microarray was performed. A total of 84 ECM proteins were tested, 20 of which were more than 2-fold upregulated in fibrotic lungs compared with normal lungs (Figure 1, Additional file 1). Among them, there were relevant proteins for the altered wound healing, such as TNC, VCAN, matrix metalloproteinases (MMPs) (MMP1, 2, 3, 7, 9, 10, 11, 12, 13, 16), secreted phosphoprotein1 (SPP1), integrin alpha7 (ITGA7) and beta3 (ITGB3). In contrast, 6 proteins were more than 2-fold downregulated, such as collagen IV alpha2 (COL4A2), laminin alpha3 (LAMA3) and member B of C-type lectin domain family 3 (CLEC3B).TNC and VCAN gene expression, as well as domain EDA of the FN, were individually analyzed in ILD patients (Figure 2A). There was a significant increase of TNC and VCAN gene expression in fibrotic lungs (IPF and cHP) compared with the normal lungs. No significant difference of FN gene expression was observed between groups (p = 0.46). Interestingly, IPF and cHP had a similar increase of TNC, VCAN and FN gene expression, with no statistical differences between both fibrotic groups. However, saHP lungs showed lower TNC and VCAN gene expression than fibrotic lungs (p = 0,032 and p = 0,082, respectively). There was no statistical association between TNC, VCAN and FN gene expression with age, sex or smoking history. On the other hand, these three glycoprotein gene expressions were statistically correlated among one another, and this relationship was stronger between TNC and VCAN (Figure 3).The upregulation of TNC synthesis in IPF and cHP was confirmed by the evaluation of TNC protein lung content by Western Blot. TNC protein expression was almost undetectable in normal or saHP lungs. IPF and cHP samples showed different levels of TNC, always higher than saHP samples (Figure 2B). No relationship was found between TNC levels and UIP radiological pattern, FVC or DLCO values at diagnosis (data not shown). Interestingly, the level of TNC in cHP samples was associated with the content of fibroblastic foci, however, there was not a clear relationship between the % of fibroblastic foci and the level of TNC in IPF lungs (p = 0,96). Furthermore, TNC levels were correlated with a significant decrease of %FVC change over 24 weeks in cHP patients (r = 0.29, p < 0.05). The highest levels of TNC also associated a %FVC decline after 24 weeks in IPF cases, however the correlation was not statistically significant (r = 3.1, p = 0.06).

### Localization of tenascin C, versican and fibronectin in lung tissue

**Figure 4:**
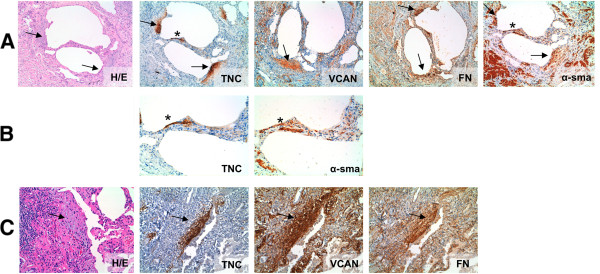
**IHC distribution of TNC, VCAN and FN in fibrotic lungs. A)** Immunohistochemical study shows a strong deposition of TNC in fibroblastic foci of IPF lungs, with more intensity in the subepithelial area. VCAN and FN are present in these fibroblastic foci, although both glycoproteins are present in other interstitial areas; **B)** A higher magnification of IPF lungs show myofibroblast rich areas (“early fibroblastic foci”, pointed with asterisk) where TNC deposition is also present; **C)** cHP lungs that showed some fibroblastic foci had TNC, VCAN and FN deposition following a similar pattern than in IPF lungs; α-sma positive cells with spindle shaped morphology localize myofibroblast. H/E: hematoxilin/eosin, TNC: tenascin C, VCAN: versican, FN: fibronectin, α-sma: alpha-smooth muscle actin.

While TNC localization in the normal lung was undetectable (Additional file 2), a well-defined pattern for TNC expression was observed in histological samples with UIP (Figure 4A-B). TNC was specifically detected in the fibroblastic foci with a protein gradient deposition and it was more intense in subepithelial areas. Moreover, in these lungs a thin subepithelial TNC expression was also observed in those regions where a few spindle shaped alpha smooth muscle actin (α-sma) positive cells (myofibroblast) were present, but no typical fibroblastic foci were observed (Figure 4B). VCAN expression was identified, associated with the elastic components in normal and pathological lungs (Additional file 2). In IPF and cHP patients, there was an increased amount of VCAN protein expression in most of fibroblastic foci. Its deposition was homogenous in the foci area (Figure 4A and 4C). FN was usually found in the inner layer of vascular walls in normal and pathological lungs, indicating uniformity throughout the lung (Additional file 2). In IPF and cHP, FN expression was also observed in fibroblastic foci, and this staining was more intensive in the subepithelial region, following a similar distribution to TNC (Figure 4A and 4C).In saHP lungs, where no FFs were present, few and specific areas of these glycoproteins were observed; TNC and FN were only localized in the largest lymphocytic aggregates with a reticular pattern. In those saHP lungs that had some histological organizing pneumonia areas, TNC was also deposited in the intraluminal polypoid fibrous structures. However, neither VCAN nor FN was present in these areas (Figure 4D).

### *In vitro* fibroblast TNC synthesis

**Figure 5:**
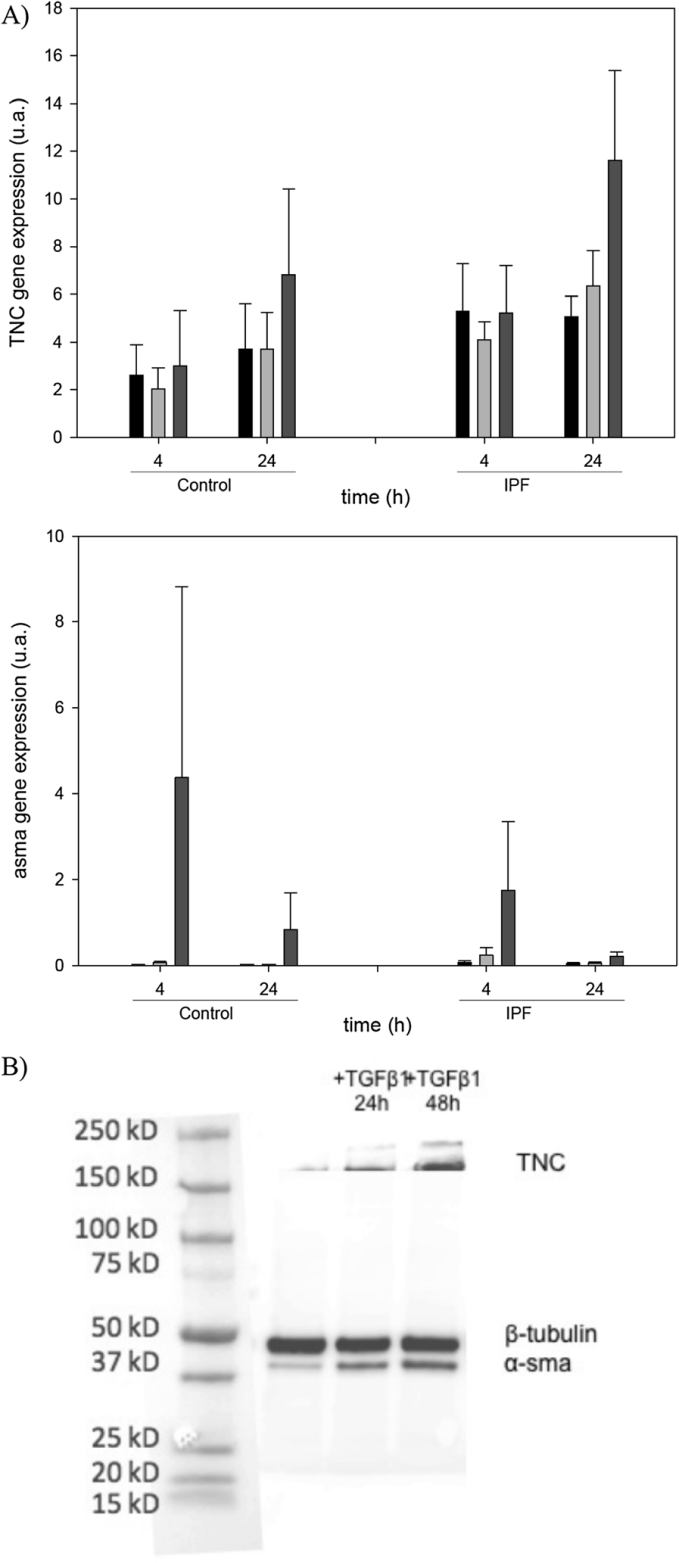
**TNC synthesis in lung fibroblasts. A)** TNC and α-sma gene expression in control and IPF fibroblasts, under different conditions; (Black) basal, (Light Gray) TNC treatment and (Black Gray) TGF-β1 treatment (at 4 and 24 h). An increase in basal and induced TNC synthesis by TGF-β1 at 24 h was observed in IPF fibroblasts. α-sma gene expression was induced by TNC and TGF-β1 at 4 h, although the effect of TNC was not significant. **B)** IPF fibroblasts after TGF-β1 treatment (24 h and 48 h) showed a progressive increase of TNC and α-sma synthesis.

In order to evaluate constitutive and induced TNC synthesis, cultured lung fibroblasts from both normal tissue and IPF patients were studied. As shown in Figure 5A, fibroblasts from IPF lungs had higher levels of TNC and α-sma gene expression compared to fibroblasts from normal lungs. TNC gene expression in both normal and fibrotic fibroblasts was increased after 24 h of TGF-β treatment, while α-sma gene expression was earlier induced. TNC induced a non-significant increase of α-sma gene expression in fibroblasts after 4 h of its addition, although no effect was observed at 24 h. Next, fibrotic fibroblast protein TNC synthesis was analyzed by Western Blot after 24 h and 48 h of TGF-β treatment (Figure 5B). An increase of TNC protein and α-sma was observed in treated cells compared to untreated ones, supporting the data found in the gene expression (Figure 5B).

## Discussion

The results of this study show that IPF lungs present a defined ECM glycoprotein pattern that differs from inflammatory interstitial (saHP) and normal lungs, but this protein profile is similar to other lung fibrotic disorders such as cHP. A better understanding of the proteome and the global gene expression of fibrotic lungs could be the clue to find specific markers involved in lung fibrotic process [14,15]. It has been reported that the ECM proteins play a key role in the maintenance of architecture and correct lung repair after any tissue injury, and an imbalance among these proteins could trigger the progression of a variety of ILD [16]. The expression of the specific proteins that form the fibrotic ECM is still a matter of discussion. In this regard, we focused on the ECM glycoproteins that were highly upregulated in fibrotic lungs compared with the normal tissue. Some of these increased ECM proteins, such as TNC and VCAN, had been involved in tissue remodeling and, specifically, in different lung diseases that imply an altered wound healing [6,8,9]. The present study demonstrates that TNC gene expression is correlated with versican and fibronectin, and its deposition is colocalized in the same fibroblastic foci, which are thought to represent ECM active areas of the altered wound healing in UIP [2]. An abnormally high deposition of TNC was previously described in lung tissue and plasma/serum of very different interstitial lung diseases; usual interstitial pneumonia (UIP), cryptogenic organizing pneumonia, sarcoidosis, HP and non-specific interstitial pneumonia [17-19]. However, this is the first time that TNC synthesis has been evaluated together with other glycoproteins in IPF lungs, and it has also been compared to other fibrotic (cHP) and non-fibrotic ILD (saHP), as well as to normal lungs. The specific and strong deposition of TNC in fibroblastic foci of UIP lungs contrasted to the small amount of TNC in inflammatory active areas of saHP, and this difference was confirmed by the study of protein and gene expression. Furthermore, our data also show that the increase of TNC was also observed in cHP, the fibrotic HP subgroup that implies high mortality, indicating that this increase of TNC is not specifically for IPF patients. Interestingly, the present results demonstrate that TNC gene expression is significantly different between cHP and saHP. This difference in the gene profile could contribute to the different outcome found between both subtypes of the same entity.

It has already been suggested that increased TNC expression in patients with UIP pattern is associated with a shortened lifespan [17]. Moreover, the relationship between TNC level and the poor outcome of other tissue remodeling entities, such as heart failure after myocardial lesion, has been previously described [20]. Despite the limitations of the present study concerning to the lack of a long-term follow-up and the total number of cases, the present results suggest a correlation between the lung levels of TNC and the progression of lung fibrosis (%FVC decline over 6 months), meanly in cHP patients. However, prospective studies with a higher number of patients are required in order to test the relationship between TNC and IPF outcome.

The relationship between VCAN and TNC has not been clearly understood yet, although a tissue co-localization of these glycoproteins has been detected in the peritumoural stroma in breast cancer [8]. However, Suwiwat and col. [8] showed that increased TNC levels, but not versican levels, were related to increased tumor size, higher grade and stage of breast carcinoma. Versican overexpression has been poorly studied in interstitial lung diseases, however its effects on motility, invasion and metastasis have been widely reported in cancer [9,21-23]. Focusing on TNC protein structure, Day and col. demonstrated that the G3 region of versican showed fairly low affinities for fibronectin type III repeats (3–5) region of TNC, so both proteins presented an interaction between each other [23]. Our results indicate a strong positive correlation between TNC and VCAN gene expression, however, the correlation between TNC and FN is not so evident. Further studies are needed to understand the exact interaction between these proteins and their effects on lung fibrosis.

Previous studies described several functional roles of TNC in the experimental fibrotic process. TNC-null mice were protected from interstitial fibrosis in bleomycin-induced lung injury, resulting from an impaired TGF-β1 responsiveness [24]. Furthermore, TNC triggers fibrin accumulation by downregulation of tissue plasminogen activator in this experimental model of lung fibrosis [25]. A possible consequence of increased TNC expression during injury is the recruitment of fibroblasts to the site of tissue damage [26]. Induced migration and adhesion in pancreatic cancer cells [6] and in α-sma cells [27] has also been described. Nagaharu and col. [28] demonstrated that this protein has also been involved in EMT-like changes in human breast cancer cells. The present results show an interstitial TNC deposition in adjacent areas to α-sma positive cells, mainly in subepithelial regions of typical fibroblastic foci and initial spaces of fibrotic activity. Moreover, an upregulation of α-sma gene expression in fibrotic fibroblasts was observed after TNC treatment. Taking together these data suggests a putative role of TNC in the formation of the fibroblastic foci, a relevant morphologic marker of the fibrotic stage.

The release and regulation of TNC depends in part on fibroblasts. Fibrotic fibroblasts constitutively synthesized more TNC than normal fibroblasts. As it was previously described for dermal fibroblasts and primary human lung fibroblasts [19,29], TGF-β1 induced synthesis of TNC, which has been shown to be mediated by SMAD or p38 MAPK pathway activation. The capacity of TGF-β1 to induce TNC synthesis has been also reported in culture alveolar epithelial cells (A549) [30]. Altogether, these data suggest that TNC upregulation could be the consequence of a pro-fibrotic molecular microenvironment after a lung injury in the active areas of altered wound healing.

## Conclusions

The ECM glycoproteins, TNC and VCAN, were highly expressed in lungs where pathologies evolved with altered wound healing, leading to fibrosis. The expression, localization and synthesis of TNC in fibrotic lungs suggest that it could be a relevant glycoprotein for fibroblastic foci activity and fibrosis progression. Further studies are needed in order to understand its effects and the possible benefits of its regulation in IPF. An alteration of ECM protein homeostasis after lung injury would play a crucial role in parenchymal lung diseases, and these proteins could be a key factor for treatment in order to inhibit this pathological process.

## Competing interests

Authors declare non-financial competing interests.

## Authors’ contributions

Conceived and designed the study: SE, VV, SS, JD and MMM. Performed the experiments and analysis: SE, AM, VV, RLL. Acquisition and interpretation of data: SE, IE, RP, AX, SS, FM, JD and MMM. All of them drafted and reviewed the manuscript. All authors read and approved the final manuscript.

## Pre-publication history

The pre-publication history for this paper can be accessed here:

http://www.biomedcentral.com/1471-2466/14/120/prepub

## Supplementary Material

Additional file 1 Complete list of genes that were up (+) or downregulated (-) in idiopathic pulmonary fibrosis patients, analysed by the cDNA microarrays. Click here for file

Additional file 2 **TNC, VCAN and FN in normal lungs.** There is no TNC in healthy lungs. VCAN and FN are found in elastic and vascular layers respectively. Click here for file
