# Supplementary material for: Lung fibrotic tenascin-C upregulation is associated with other extracellular matrix proteins and induced by TGFβ1
Source: BMC Pulm Med. 2014 Jul 26;14:120. doi: 10.1186/1471-2466-14-120 (PMC4123829; doi:10.1186/1471-2466-14-120)
Supplement: Additional file 1 — Complete list of genes that were up (+) or downregulated (-) in idiopathic pulmonary fibrosis patients, analysed by the cDNA microarrays. [file 1471-2466-14-120-S1.pdf]

Additional file 1: Complete list of genes that were up (+) or downregulated (-) in idiopathic pulmonary fibrosis patients, analysed by the cDNA microarrays.

			2-ΔCt		Fold	
**Symbol**	**UniGene**	**RefSeq**	**IPF**	**Control**	**IPF/control**	**Description**
ADAMTS1	Hs.643357	NM_006988	0,06	0,06	-1,16	ADAM metallopeptidase thrombospondin type 1, 1
ADAMTS13	Hs.131433	NM_139025	0,00	0,00	-1,08	ADAM metallopeptidase thrombospondin type 1, 13
ADAMTS8	Hs.271605	NM_007037	0,02	0,02	-1,20	ADAM metallopeptidase thrombospondin type 1, 8
CD44	Hs.502328	NM_000610	0,41	0,41	-1,01	CD44 molecule (Indian blood group)
CDH1	Hs.461086	NM_004360	0,10	0,12	-1,13	Cadherin 1, type 1, E-cadherin (epithelial)
CNTN1	Hs.143434	NM_001843	0,01	0,01	1,07	Contactin 1
COL11A1	Hs.523446	NM_080629	0,00	0,00	-3,37	Collagen, type XI, alpha 1
COL12A1	Hs.101302	NM_004370	0,10	0,08	1,22	Collagen, type XII, alpha 1
COL14A1	Hs.409662	NM_021110	0,20	0,07	2,92	Collagen, type XIV, alpha 1
COL15A1	Hs.409034	NM_001855	0,06	0,02	3,15	Collagen, type XV, alpha 1
COL16A1	Hs.368921	NM_001856	0,03	0,03	1,21	Collagen, type XVI, alpha 1
COL1A1	Hs.172928	NM_000088	0,54	0,29	1,88	Collagen, type I, alpha 1
COL4A2	Hs.508716	NM_001846	0,28	0,61	-2,14	Collagen, type IV, alpha 2
COL5A1	Hs.210283	NM_000093	0,08	0,06	1,50	Collagen, type V, alpha 1
COL6A1	Hs.474053	NM_001848	0,23	0,16	1,45	Collagen, type VI, alpha 1
COL6A2	Hs.420269	NM_001849	0,15	0,10	1,46	Collagen, type VI, alpha 2
COL7A1	Hs.476218	NM_000094	0,01	0,00	5,82	Collagen, type VII, alpha 1
COL8A1	Hs.654548	NM_001850	0,01	0,00	1,74	Collagen, type VIII, alpha 1
VCAN	Hs.643801	NM_004385	0,05	0,02	2,13	Versican
CTGF	Hs.591346	NM_001901	0,13	0,28	-2,08	Connective tissue growth factor
CTNNA1	Hs.534797	NM_001903	0,21	0,26	-1,24	Catenin (cadherin-associated prot.), alpha 1, 102kDa
CTNNB1	Hs.476018	NM_001904	0,20	0,23	-1,14	Catenin (cadherin-associated prot.), beta 1, 88kDa
CTNND1	Hs.166011	NM_001331	0,15	0,26	-1,71	Catenin (cadherin-associated prot.), delta 1
CTNND2	Hs.314543	NM_001332	0,00	0,00	-4,10	Catenin (cadherin-associated prot.), delta 2
ECM1	Hs.81071	NM_004425	0,02	0,01	1,47	Extracellular matrix protein 1
FN1	Hs.203717	NM_002026	2,70	2,19	1,23	Fibronectin 1
HAS1	Hs.57697	NM_001523	0,00	0,00	-1,02	Hyaluronan synthase 1
CAM1	Hs.643447	NM_000201	0,25	0,28	-1,12	Intercellular adhesion molecule 1
TGA1	Hs.644352	NM_181501	0,15	0,25	-1,59	Integrin, alpha 1
TGA2	Hs.482077	NM_002203	0,02	0,02	1,25	Integrin, alpha 2 (CD49B, alpha 2 subunit VLA-2 R)
TGA3	Hs.265829	NM_002204	0,07	0,10	-1,55	Integrin, alpha 3 (CD49C, alpha 3 subunit VLA-3R)
TGA4	Hs.694732	NM_000885	0,05	0,04	1,16	Integrin, alpha 4 (CD49D, alpha 4 subunit VLA-4R)
TGA5	Hs.505654	NM_002205	0,05	0,07	-1,45	Integrin, alpha 5 (fibronectin receptor, alpha polyp.)
TGA6	Hs.133397	NM_000210	0,11	0,17	-1,63	Integrin, alpha 6
TGA7	Hs.524484	NM_002206	0,01	0,00	2,19	Integrin, alpha 7
TGA8	Hs.171311	NM_003638	0,08	0,10	-1,18	Integrin, alpha 8
TGAL	Hs.174103	NM_002209	0,02	0,02	-1,01	Integrin, alpha L
TGAM	Hs.172631	NM_000632	0,02	0,02	1,43	Integrin, alpha M
TGAV	Hs.436873	NM_002210	0,10	0,13	-1,27	Integrin, alpha V
TGB1	Hs.643813	NM_002211	0,47	0,54	-1,13	Integrin, beta 1
TGB2	Hs.375957	NM_000211	0,22	0,29	-1,27	Integrin, beta 2
TGB3	Hs.218040	NM_000212	0,01	0,00	2,12	Integrin, beta 3
TGB4	Hs.632226	NM_000213	0,02	0,02	1,17	Integrin, beta 4
TGB5	Hs.536663	NM_002213	0,08	0,07	1,05	Integrin, beta 5
KAL1	Hs.521869	NM_000216	0,08	0,15	-1,72	Kallmann syndrome 1 sequence
LAMA1	Hs.270364	NM_005559	0,00	0,00	1,36	Laminin, alpha 1
LAMA2	Hs.200841	NM_000426	0,05	0,06	-1,12	Laminin, alpha 2
LAMA3	Hs.436367	NM_000227	0,03	0,08	-2,68	Laminin, alpha 3
LAMB1	Hs.650585	NM_002291	0,11	0,15	-1,27	Laminin, beta 1

LAMB3	Hs.497636	NM_000228	0,04	0,04	1,00	Laminin, beta 3
LAMC1	Hs.609663	NM_002293	0,20	0,25	-1,29	Laminin, gamma 1 (formerly LAMB2)
MMP1	Hs.83169	NM_002421	0,06	0,00	26,62	Matrix metallopeptidase 1 (interstitial collagenase)
MMP10	Hs.2258	NM_002425	0,01	0,00	2,26	Matrix metallopeptidase 10 (stromelysin 2)
MMP11	Hs.143751	NM_005940	0,01	0,01	2,51	Matrix metallopeptidase 11 (stromelysin 3)
MMP12	Hs.1695	NM_002426	0,01	0,00	15,66	Matrix metallopeptidase 12 (macrophage elastase)
MMP13	Hs.2936	NM_002427	0,01	0,00	26,33	Matrix metallopeptidase 13 (collagenase 3)
MMP14	Hs.2399	NM_004995	0,03	0,03	1,22	Matrix metallopeptidase 14 (membrane-inserted)
MMP15	Hs.80343	NM_002428	0,00	0,00	-1,45	Matrix metallopeptidase 15 (membrane-inserted)
MMP16	Hs.546267	NM_005941	0,01	0,00	3,82	Matrix metallopeptidase 16 (membrane-inserted)
MMP2	Hs.513617	NM_004530	0,27	0,14	2,00	Matrix metallopeptidase 2 (gelatinase A)
MMP3	Hs.375129	NM_002422	0,00	0,00	4,98	Matrix metallopeptidase 3 (stromelysin 1)
MMP7	Hs.2256	NM_002423	0,23	0,01	18,78	Matrix metallopeptidase 7 (matrilysin, uterine)
MMP8	Hs.161839	NM_002424	0,00	0,00	4,72	Matrix metallopeptidase 8 (neutrophil collagenase)
MMP9	Hs.297413	NM_004994	0,11	0,01	8,98	Matrix metallopeptidase 9 (gelatinase B)
NCAM1	Hs.503878	NM_000615	0,01	0,00	2,26	Neural cell adhesion molecule 1
PECAM1	Hs.514412	NM_000442	0,08	0,10	-1,14	Platelet/endothelial cell adhesion molecule
SELE	Hs.89546	NM_000450	0,00	0,00	2,11	Selectin E
SELL	Hs.82848	NM_000655	0,02	0,01	1,32	Selectin L
SELP	Hs.73800	NM_003005	0,03	0,04	-1,11	Selectin P
SGCE	Hs.371199	NM_003919	0,06	0,11	-1,73	Sarcoglycan, epsilon
SPARC	Hs.111779	NM_003118	0,74	1,05	-1,41	Secreted protein, acidic, cysteine-rich (osteonectin)
SPG7	Hs.185597	NM_003119	0,02	0,03	-1,36	Spastic paraplegia 7
SPP1	Hs.313	NM_000582	0,47	0,02	31,33	Secreted phosphoprotein 1
TGFBI	Hs.369397	NM_000358	0,19	0,10	1,92	Transforming growth factor, beta-induced
THBS1	Hs.164226	NM_003246	0,10	0,08	1,28	Thrombospondin 1
THBS2	Hs.371147	NM_003247	0,10	0,06	1,64	Thrombospondin 2
THBS3	Hs.169875	NM_007112	0,01	0,00	1,49	Thrombospondin 3
TIMP1	Hs.522632	NM_003254	1,23	1,03	1,19	TIMP metallopeptidase inhibitor 1
TIMP2	Hs.633514	NM_003255	0,24	0,32	-1,36	TIMP metallopeptidase inhibitor 2
TIMP3	Hs.644633	NM_000362	0,00	0,00	-1,64	TIMP metallopeptidase inhibitor 3
CLEC3B	Hs.476092	NM_003278	0,08	0,21	-2,43	C-type lectin domain family 3, member B
TNC	Hs.143250	NM_002160	0,24	0,06	2,88	Tenascin C
VCAM1	Hs.109225	NM_001078	0,04	0,04	1,05	Vascular cell adhesion molecule 1
VTN	Hs.2257	NM_000638	0,00	#N/D	N/A	Vitronectin
