# Supplementary figures and images for: Lung fibrotic tenascin-C upregulation is associated with other extracellular matrix proteins and induced by TGFβ1
Source: BMC Pulm Med. 2014 Jul 26;14:120. doi: 10.1186/1471-2466-14-120 (PMC4123829; doi:10.1186/1471-2466-14-120)

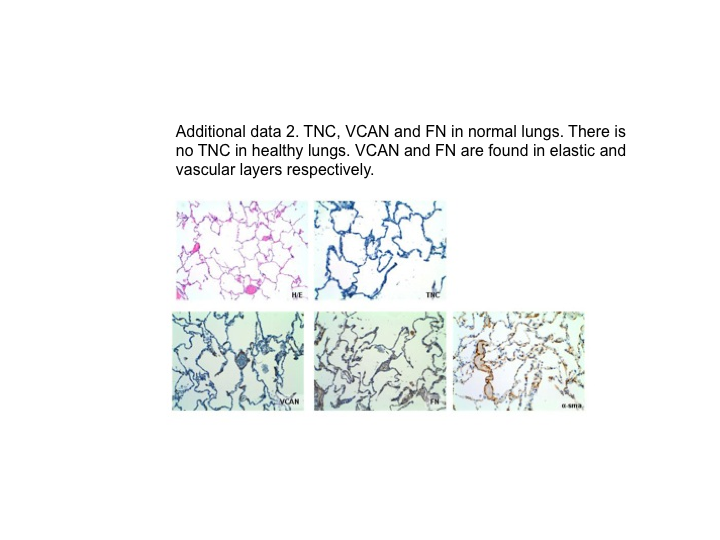

Supplement: Additional file 2 — TNC, VCAN and FN in normal lungs. There is no TNC in healthy lungs. VCAN and FN are found in elastic and vascular layers respectively. [file 1471-2466-14-120-S2.png]
